# Factors Associated with ICU Admission following Blunt Chest Trauma

**DOI:** 10.1155/2016/3257846

**Published:** 2016-12-01

**Authors:** Andrea Bellone, Ilaria Bossi, Massimiliano Etteri, Francesca Cantaluppi, Paolo Pina, Massimo Guanziroli, AnnaMaria Bianchi, Giovanni Casazza

**Affiliations:** ^1^Emergency Ward, Niguarda Hospital, Milan, Piazza Ospedale Maggiore 3, 20162 Milano, Italy; ^2^Emergency Ward, Azienda Ospedaliera Sant'Anna di Como, Via Ravona 19, 22020 Como, Italy; ^3^Dipartimento di Scienze Biomediche e Cliniche L. Sacco, Università degli Studi di Milano, Via GB Grassi 74, 20157 Milano, Italy

## Abstract

*Background*. Blunt chest wall trauma accounts for over 10% of all trauma patients presenting to emergency departments worldwide. When the injury is not as severe, deciding which blunt chest wall trauma patients require a higher level of clinical input can be difficult. We hypothesized that patient factors, injury patterns, analgesia, postural condition, and positive airway pressure influence outcomes.* Methods*. The study population consisted of patients hospitalized with at least 3 rib fractures (RF) and at least one pulmonary contusion and/or at least one pneumothorax lower than 2 cm.* Results*. A total of 140 patients were retrospectively analyzed. Ten patients (7.1%) were admitted to intensive care unit (ICU) within the first 72 hours, because of deterioration of the clinical conditions and gas exchange with worsening of chest X-ray/thoracic ultrasound/chest computed tomography. On univariable analysis and multivariable analysis, obliged orthopnea (*p* = 0.0018) and the severity of trauma score (*p* < 0.0002) were associated with admission to ICU.* Conclusions*. Obliged orthopnea was an independent predictor of ICU admission among patients incurring non-life-threatening blunt chest wall trauma. The main therapeutic approach associated with improved outcome is the prevention of pulmonary infections due to reduced tidal volume, namely, upright postural condition and positive airway pressure.

## 1. Introduction

Blunt chest wall trauma accounts for over 10% of all trauma patients presenting to emergency departments worldwide [1]. Research has highlighted significant morbidity and mortality for the blunt chest wall trauma patient, with reported mortality ranging from 4 to 20% [1, 2]. The patient with severe thoracic injuries will be managed in the emergency department (Dpt) by trauma and various surgical teams and intervention is dictated by the resuscitation protocol of the department [3]. Disposition of chest injury patients from the emergency department is therefore straightforward when the patient requires immediate surgery or supportive mechanical ventilation [3]. When the injury is not as severe or associate injuries are not present or are minor, deciding which blunt chest wall trauma patients require a higher level of clinical input can be difficult. Clinical symptoms are not considered an accurate predictor of outcome following non-life-threatening blunt chest wall trauma [4]. The aim of this study was to identify the risk factors for admission to the intensive care unit in non-life-threatening patients with blunt chest trauma admitted to the emergency medicine ward and immediately submitted to a strategy that included positive airway pressure, upright position, and pain-control by pharmacologic therapy.

## 2. Materials and Methods

### 2.1. Participants and Study Design

This study was performed in a busy, level 1 trauma center. Between January 2013 and December 2014, 140 patients with non-life-threatening blunt chest wall trauma were reviewed retrospectively. Approval was obtained from the institutional review board. All injured patients received a standardized examination including bedside chest and pelvis radiography, abdominal and thoracic ultrasound (extended focused assessment with sonography for trauma), and computed tomography of head, spine, chest, abdomen, and pelvis. As a part of the routine, plain radiography of the chest was taken 48 hours after admission in the emergency ward.

Patients were enrolled in the study according to the criteria listed as follows.

*Inclusion Criteria*. They include the following: (1) 18 years of age or more; (2) more than three rib fractures and/or lung contusion and/or pneumothorax and/or sternal fracture; (3) admission to the hospital within 24 hours after injury. 

*Exclusion Criteria*. They include the following: (1) chest wall trauma score more than 7 [5]; (2) pressure of arterial oxygen/fractional inspired oxygen concentration (PaO_2_/FiO_2_) < 250; (3) the need for vasopressor agents; (4) the need for immediate intubation and mechanical ventilation; (5) the need for pneumothorax drainage; (6) severe traumatic injury other than blunt chest wall trauma.

All patients were submitted to a standardized therapeutic program: (1) keeping the posture at 45° or more; (2) cycle of positive airway pressure by Continuous Positive Airway Pressure (CPAP) trial (three hours every six hours) for the first 24 hours: in the next period, patients were encouraged to blow through a tube with 10 cm of water for at least 5 minutes every three hours; (3) patient-controlled analgesia by 200 mg of tapentadol a day; if not effective as reported by numerical rating scale (NRS) more than 7, we used transcutaneous fentanyl (50 mcg/hour) as rescue therapy.

The aim of our study was to identify the risk factors for the admission to the ICU. The decision to admit patients to the ICU was made by the senior emergency, surgical, and intensive care/anaesthetic doctor. We used as criteria for improving our decision the following: PaO_2_/FiO_2_ < 250; the need for vasopressor agents; and the need for immediate intubation and mechanical ventilation.

### 2.2. Statistical Analysis

Descriptive analyses were performed by calculating mean (±standard deviation, SD) or median (interquartile range, IQR), as appropriate, for quantitative continuous variables. Categorical variables were reported as count (percentage).

Univariate and multivariate logistic regression analyses were performed [6] to assess the effect of age (dichotomized, >65 years versus ≤65 years), chest wall score, injury score, SpO_2_, number of ribs fractures, chest contusion, and obliged orthopnea on the risk of being admitted to the ICU. Only those variables that were statistically significant in univariate models were considered in multivariate analysis.

Results were reported as odds ratios (OR) with 95% confidence intervals (CI).

The *c*-statistic, ranging from 0.5 (chance prediction) to 1.0 (perfect prediction of the events), was used to assess the predictive ability of the logistic models.

*p* values less than 0.05, two-tailed, were considered statistically significant. All of the statistical analyses were performed using SAS statistical software (release 9.4, SAS Institute Inc., Cary, NC, USA).

## 3. Results

**Figure 1:**
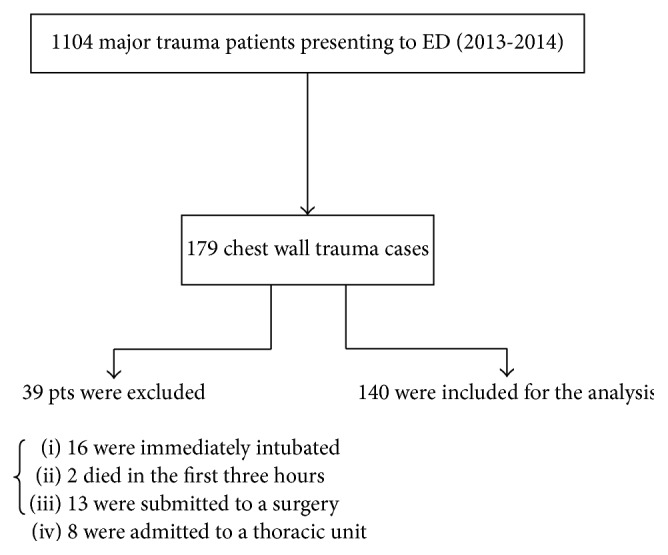
Flow diagram.

During the study period, 1104 consecutive patients were admitted to our hospital because of major trauma. Of these, 179 patients presented mainly chest wall trauma. Thirty-nine patients were excluded from the study (16 patients were immediately intubated, two patients died in the first three hours, 13 patients were sent to the operating surgery, and 8 patients were admitted to the thoracic unit) (Figure 1). One hundred forty met the inclusion criteria for our study. Of all included patients 80% were victims of high energy chest trauma, due to car or motorcycle crashes or falls from large height, while the 20% were victims of low energy trauma with minor car crashes or domestic accidents.

All patients were submitted to patient-controlled analgesia, upright postural condition, and positive airway pressure in Dpt of emergency (Table 1). Only 11 patients had to prolong CPAP treatment for 36 hours because of the respiratory distress persistence.

Ten of these patients (7.1%) went on to require ICU admission within the first 72 hours, because of a deterioration of the clinical conditions and gas exchange. For all patients were performed chest US and chest XR and in 7 cases they showed an enlargement of pulmonary consolidations confirmed with CT scan.

The characters of these patients in terms of trauma severity were not significantly different compared with the remaining patients (Table 3). None of these patients died.

The 130 patients were discharged from the emergency ward and the medium length of stay in hospital was 6.4 days. No patients were admitted to our hospital in the next two months.

The mean injury severity score was 15 [7]. The mean chest wall score was 4, 7 [8]. The median number of fractured ribs was 4 (IQR 3–6). Oxygenation as measured by arterial oxygen tension (PaO_2_)/inspiratory oxygen fraction (FiO_2_) and respiratory function as measured by respiratory rate, serum pH, pCO_2_, and bicarbonate before the initial management are presented in Table 2.

Tapentadol was used in 89% of patients. Only 11% of patients needed transcutaneous fentanyl because of numeric rating scale (NRS) more than 7.

At univariate analysis, the injury score and obliged orthopnea were the only statistically significant factors for the prediction of the admission to the ICU (Table 2). This result was confirmed in the multivariate analysis (injury score, OR = 1.17, 95% CI 1.06 to 1.30, and *p* = 0.0018; obliged orthopnea OR = 20.3, 95% CI 4.08 to 101.4, and *p* = 0.0002). The multivariate model containing the injury score and obliged orthopnea showed an overall good predictive ability (*c*-statistic = 0.914).

Following multivariate analysis, the obliged postural condition was a significant factor associated with ICU requirement.

## 4. Discussion

As no current guidelines exist for the management of this patient group, recognition of the high risk patient in the ED is not always straightforward due to the nature of the injury and its recovery phase. The blunt chest wall trauma patient who can walk into the ED with no immediate life-threatening injury will commonly develop complications up to 72 h or more after injury, which may also prove life-threatening [9, 10]. An understanding of the risk factors for development of late complications in blunt chest wall trauma patient requiring the admission to the ICU could assist in the accurate risk stratification of this patient group in the ED and thus improve outcomes.

Our study has three strengths: our approach was aggressive. We start pain management with pharmacologic therapy. Our decision was in favour of the pharmacological pain-control because two previous studies showed that the insertion of intercostal catheters was significantly associated with morbidity [10, 11]; secondly, all patients were immediately submitted to a positive airway pressure by mask or by a tube. It is well known that, in chest trauma, a lung lesion such as pulmonary contusion or pneumothorax and/or thoracic injury can promote systemic inflammatory activation and consequently an acute respiratory failure due to alveolar collapse and impaired fluid clearance [12]. Recently a systematic review and meta-analysis suggested that noninvasive ventilation could be useful in the management of acute respiratory failure due to chest trauma [13]; third, to keep an obliged posture at, at least, 45 degrees means to improve the ventilation/perfusion ratio by increasing the functional residual capacity with a better ventilation distribution towards more perfused lung areas [14].

A previous study that analyzed factors associated with survival following blunt chest trauma in older patients showed that age and injury severity score were independent predictors of survival [15]. On the opposite, another study showed that the risk factors for the development of complications in the recovery phase following blunt chest wall trauma were a patient age of 65 years or more, three or more rib fractures, chronic lung disease or cardiovascular disease, the use of preinjury anticoagulants, and oxygen saturation level in the ED of less than 90% [16].

In our study, two factors were associated with patients' admission to the ICU from the emergency ward. Patients with high injury score and patients with obliged orthopnea were at high risk of admission to the ICU. These two factors showed an excellent ability in predicting admission to the ICU, as shown by the high value of the *c*-statistic (0.91).

In particular data regarding obliged orthopnea testify that the topics of chest trauma management should be based on three principles (pain-control, positive airway pressure, and posture) serving all together to prevent atelectasis and lung infection. Age, oxygenation, number of rib fractures, comorbidity, and preinjury anticoagulants do not seem to affect patients' outcome in non-life-threatening blunt chest wall trauma. Data should be confirmed with larger and different clinical records.

This study has limitations. Retrospective data were used. Such analyses are prone to selection bias and, in general, are more suitable for developing study questions rather than answering scientific questions. Secondly, the analysis of patients was performed in only one center. Third, the number of studied patients is limited. Since we observed only ten events, the results obtained with our multivariate model might be unstable. A larger multicenter study is needed to confirm our results.

## Figures and Tables

**Table 1:** Characteristics of patients on admission.

Age (years)	Mean (range)	66 (52–76)

Sex	F	41 (29.3%)
	M	99 (70.7%)

Charlson comorbidity index	0–3	81 (57,8%)
	>3	59 (42.2%)

SpO_2_	Mean (range)	96% (94%–98%)

Number of ribs fractures	0–3	63 (45%)
	>3	77 (55%)

PNX	Yes	48 (34.3%)
	No	92 (65.7%)

Number of chest contusions	0	103 (73.6%)
	1	23 (16.4%)
	≥2	14 (10.0%)

Head injury	Yes	39 (27.9%)
	No	101 (72.1%)

Hipbone	Yes	4 (2.9%)
	No	136 (97.1%)

Spine fracture	Yes	21 (15.0%)
	No	119 (85.5%)

Previous anticoagulant therapy	Yes	11 (7.9%)
	No	129 (92.1%)

Clavicula/sterna/scapula fractures	Yes	38 (27.1%)
	No	102 (72.9%)

**Table 2:** Statistical analysis.

Variable	Univariate model		Multivariate model	
Age > 65 yrs	1.25 (0.38 to 4.07)	0.7162	—	
Chest wall score	1.10 (0.72 to 1.67)	0.6648	—	
Injury score	1.16 (1.07 to 1.25)	0.0002	1.17 (1.06 to 1.30)	0.0018
SpO_2_	0.90 (0.80 to 1.01)	0.0818	—	
Number of ribs fractures	1.18 (0.96 to 1.46)	0.1230	—	
Chest contusion	2.14 (0.64 to 7.23)	0.2190	—	
Fractures with immobilization	22.6 (5.50 to 92.9)	<0.0001	20.3 (4.08 to 101.4)	0.0002

*c*-statistic for multivariate model: 0.914.

**Table 3:** Patients admitted to ICU.

Age (years)	Mean (range)	71 (58–76)

Sex	F	5 (50%)
	M	5 (50%)

Charlson comorbidity index	0–3	8 (80%)
	>3	2 (20%)

SpO_2_		93,8% (75%–100%)

Number ribs fractures	0–3	3 (30%)
	>3	7 (70%)

PNX	Yes	6 (60%)
	No	4 (40%)

Number chest contusions	0	4 (40%)
	1	1 (10%)
	≥2	5 (50%)

Head injury	Yes	3 (30%)
	No	7 (70%)

Hipbone fracture	Yes	2 (20%)
	No	8 (80%)

Spine fracture	Yes	3 (30%)
	No	7 (70%)

Previous anticoagulant therapy	Yes	1 (10%)
	No	9 (90%)

Clavicula/sternal/scapula fractures	Yes	2 (20%)
	No	8 (80%)

Fracture with immobilization	yes	8 (80%)
	No	2 (20%)
